# Crystal structure of 1,3-bis­(4-hexyl-5-iodo­thio­phen-2-yl)-4,5,6,7-tetra­hydro-2-benzo­thio­phene

**DOI:** 10.1107/S1600536814019667

**Published:** 2014-09-27

**Authors:** Julian Linshoeft, Christian Näther, Anne Staubitz

**Affiliations:** a Otto-Diels-Institut für Organische Chemie, Christian-Albrechts-Universität Kiel, Otto-Hahn-Platz 4, 24118 Kiel, Germany; b Institut für Anorganische Chemie, Christian-Albrechts-Universität Kiel, Max-Eyth-Str. 2, 24118 Kiel, Germany

**Keywords:** crystal structure, thio­phene, 2-benzo­thio­phene, monomer

## Abstract

In the crystal structure of the title compound, C_28_H_36_I_2_S_3_, a terthio­phene monomer, the central thio­phene unit is arranged *anti*-coplanar to the two outer thio­phene rings. There are two crystallographically independent mol­ecules in the asymmetric unit, which show different conformations. In one mol­ecule, the dihedral angles between the inner and the two outer thiophene rings are 15.7 (3) and 3.47 (3)°, whereas these values are 4.2 (3) and 11.3 (3)° for the second mol­ecule. Differences are also found in the arrangement of the hexyl chains: in one of the two molecules, both chains are nearly in plane to the central moiety, whereas in the second molecule, only one chain is in plane and the other one is nearly perpendicular to the central moiety. Some of the C atoms are disordered and were refined using a split model with occupancy ratios of 0.65:0.35 and 0.70:0.30 in the two mol­ecules.

## Related literature   

For the synthesis of the starting materials, as well as the synthesis, crystal structure and polymerization of a similar thio­phene-flanked stannole monomer, see: Linshoeft *et al.* (2014 ▶). For typical bond lengths of other thio­phene rings, see: Chaloner *et al.* (1997 ▶). For more information about thio­phenes as important heterocycles for semiconducting materials, see: Thompson & Fréchet (2007 ▶); Mishra *et al.* (2009 ▶).
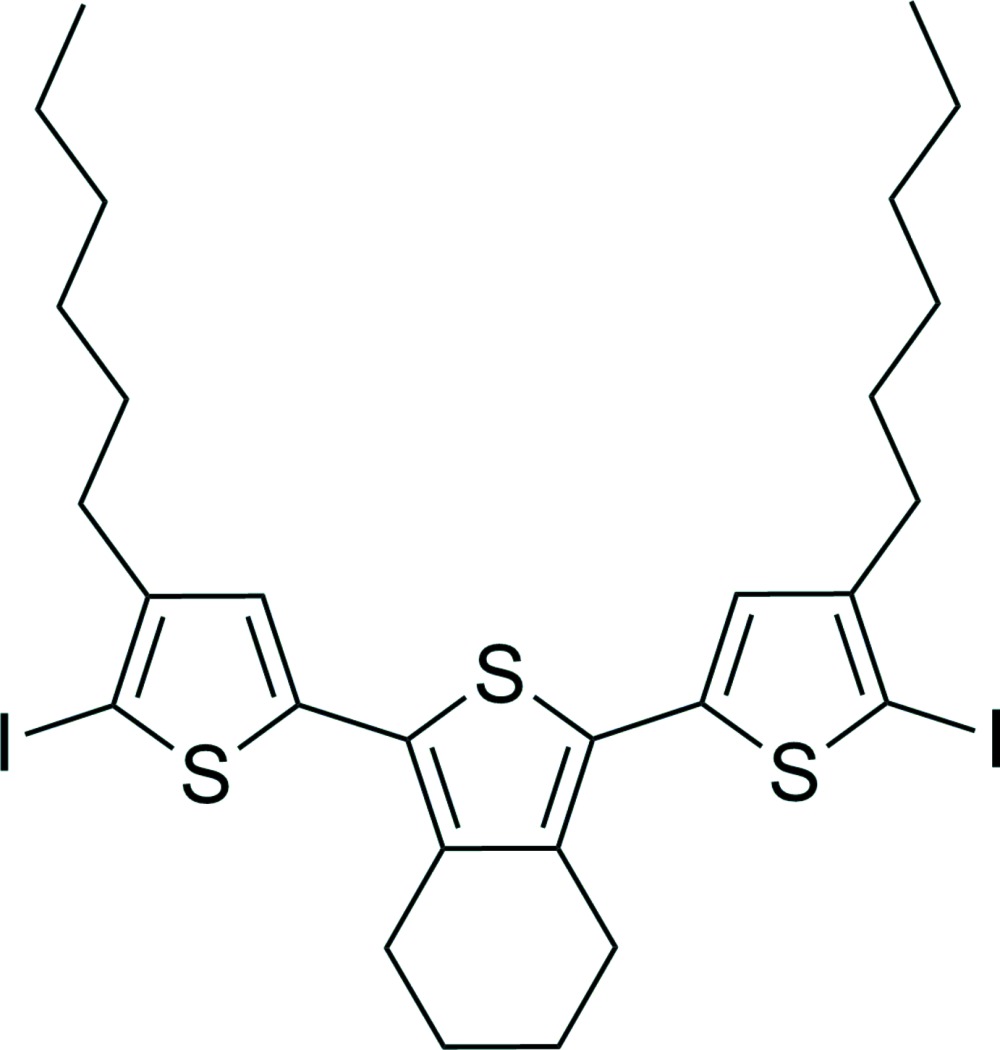


## Experimental   

### Crystal data  

C_28_H_36_I_2_S_3_
*M* *_r_* = 722.55
Triclinic, 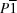

*a* = 13.4491 (4) Å
*b* = 14.9488 (5) Å
*c* = 16.1260 (5) Å
α = 73.387 (2)°
β = 71.208 (2)°
γ = 77.794 (3)°
*V* = 2915.60 (16) Å^3^
*Z* = 4
Mo *K*α radiation
μ = 2.39 mm^−1^
*T* = 200 K
0.16 × 0.10 × 0.08 mm

### Data collection  

Stoe IPDS-1 diffractometer
Absorption correction: numerical (*X-SHAPE* and *X-RED32*; Stoe & Cie, 2008 ▶) *T*_min_ = 0.748, *T*_max_ = 0.815
26585 measured reflections
12585 independent reflections
9932 reflections with *I* > 2σ(*I*)
*R*_int_ = 0.038

### Refinement  

*R*[*F* ^2^ > 2σ(*F* ^2^)] = 0.034
*wR*(*F* ^2^) = 0.085
*S* = 0.98
12585 reflections
640 parameters
3 restraints
H-atom parameters constrained
Δρ_max_ = 1.01 e Å^−3^
Δρ_min_ = −0.81 e Å^−3^

### 

Data collection: *X-AREA* (Stoe & Cie, 2008 ▶); cell refinement: *X-AREA*; data reduction: *X-AREA*; program(s) used to solve structure: *SHELXS97* (Sheldrick, 2008 ▶); program(s) used to refine structure: *SHELXL97* (Sheldrick, 2008 ▶); molecular graphics: *XP* in *SHELXTL* (Sheldrick, 2008 ▶); software used to prepare material for publication: *publCIF* (Westrip, 2010 ▶).

## Supplementary Material

Crystal structure: contains datablock(s) I, New_Global_Publ_Block. DOI: 10.1107/S1600536814019667/bt6994sup1.cif

Structure factors: contains datablock(s) I. DOI: 10.1107/S1600536814019667/bt6994Isup2.hkl

Supporting information file. DOI: 10.1107/S1600536814019667/bt6994Isup3.cml

**Fig. 1.**
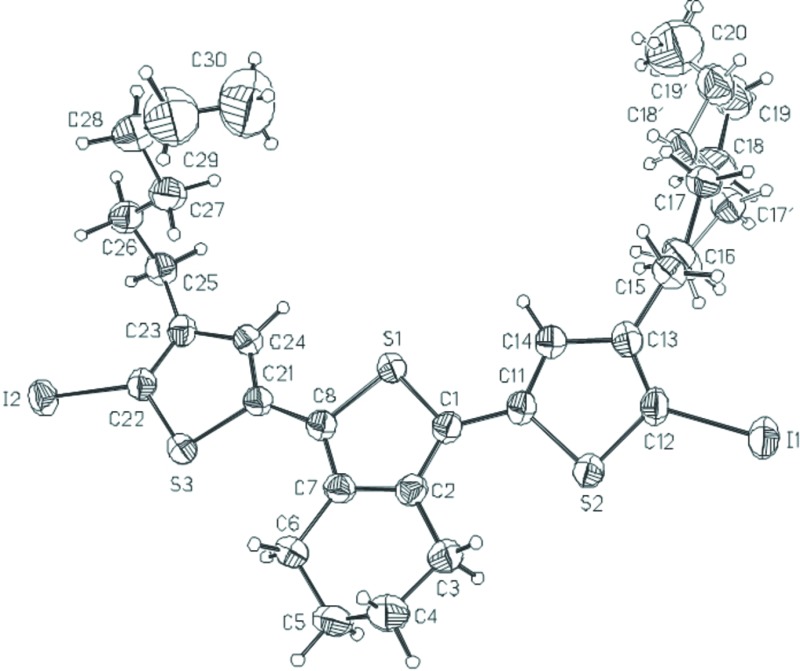
: Molecular structure of the first of the two crystallographically independent molecules with labelling and displacement ellipsoids drawn at the 50% probability level. The minor occupied atoms of the disordered sites are drawn with open bonds.

. DOI: 10.1107/S1600536814019667/bt6994fig1.tif
Mol­ecular structure of the first of the two crystallographically independent mol­ecules with labelling and displacement ellipsoids drawn at the 50% probability level. The minor occupied atoms of the disordered sites are drawn with open bonds.

**Fig. 2.**
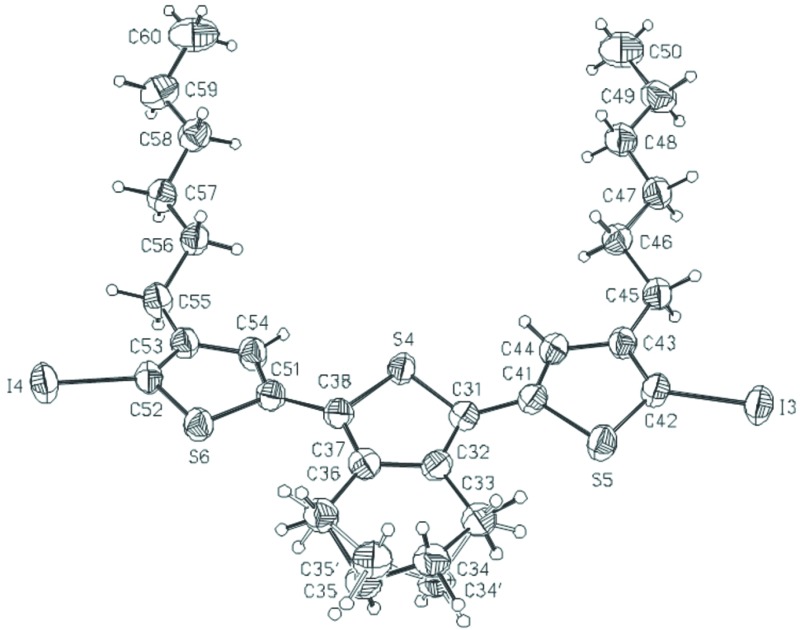
: Molecular structure of the second of the two crystallographically independent molecules with labeling and displacement ellipsoids drawn at the 50% probability level·The minor occupied atoms of the disordered sites are drawn with open bonds.

. DOI: 10.1107/S1600536814019667/bt6994fig2.tif
Mol­ecular structure of the second of the two crystallographically independent mol­ecules with labeling and displacement ellipsoids drawn at the 50% probability level·The minor occupied atoms of the disordered sites are drawn with open bonds.

CCDC reference: 1021995

Additional supporting information:  crystallographic information; 3D view; checkCIF report

## supplementary crystallographic information

### S1.1. Synthesis and crystallization

1,8-Bis(4-hexyl-5-iodo­thio­phen-2-yl)o­cta-1,7-diyne and Cp_2_Zr(pyr)(Me_3_SiCCSiMe_3_) were prepared as previously described by Linshoeft *et al.* (2014). CuCl (99.995+ %) was bought from Alfa Aesar, S_2_Cl_2_ (98 %) from VWR. Toluene was dried over sodium with benzo­phenone as an indicator, degassed by freeze-pump-thaw technique and stored over 3Å molecular sieve in a glovebox.

1,8-Bis(4-hexyl-5-iodo­thio­phen-2-yl)o­cta-1,7-diyne (716 mg, 1.04 mmol) and Cp_2_Zr(pyr)(Me_3_SiCCSiMe_3_) (513 mg, 1.09 mmol) were dissolved in toluene (10 mL) and the dark red solution was stirred for 18 h at 20 °C under nitro­gen. CuCl (10.3 mg, 104 µmol) and toluene (2 mL) were added in a glovebox under an atmosphere of nitro­gen and the reaction mixture was cooled to 0 °C. S_2_Cl_2_ (147 mg, 1.09 mmol) was added dropwise within 1 min, resulting in an immediate color change from dark red to orange. The reaction mixture was stirred for 1 h at 0 °C, then for another 2 h at 20 °C, before it was quenched with water (50 mL). The aqueous layer was extracted with cyclo­hexane (3 x 80mL). The combined organic layers were dried over magnesium sulfate. The volatiles were removed *in vacuo* and the crude product was filtered over a short plug of silica (cyclo­hexane; 3 x 4 cm). After removal of the volatiles, the orange oil was purified by column chromatography (*n*-hexane; *R_f_* = 0.62) to obtain 395 mg (53 %) of a yellow oil that crystallized after 14 h at 7 °C.

Single crystals could be obtained from a saturated solution (*n*-pentane) at 7°C.

^1^H NMR (500 MHz, CDCl_3_): *δ* = 6.79 (s, 2 H, Tph-*H*), 2.72 – 2.81 (m, 4 H, -C*H*_2_CH_2_CH_2_C*H*_2_-), 2.52 (t, ^3^*J* = 7.7 Hz, 4 H, -C*H*_2_(CH_2_)_4_CH_3_), 1.81 – 1.76 (m, 4 H, -CH_2_C*H*_2_C*H*_2_CH_2_-), 1.62 – 1.55 (m, 4 H, -CH_2_C*H*_2_(CH_2_)_3_CH_3_), 1.38 – 1.30 (m, 12 H, -(CH_2_)_2_C*H*_2_C*H*_2_C*H*_2_CH_3_), 0.87 – 0.80 (m, 6 H, -(CH_2_)_5_C*H*_3_) ppm. ^13^C NMR (126 MHz, CDCl_3_): *δ* = 147.6 (Tph-*C*), 141.0 (Tph-*C*), 136.2 (Tph-*C*), 128.5 (Tph-*C*), 125.3 (Tph-*C*H), 73.7 (Tph-*C*), 32.3 (-*C*H_2_(CH_2_)_4_CH_3_), 31.6 (*n*Hex-*C*), 30.0 (-CH_2_*C*H_2_(CH_2_)_3_CH_3_), 28.9 (*n*Hex-*C*), 27.1 (-*C*H_2_CH_2_CH_2_*C*H_2_-), 22.9 (-CH_2_*C*H_2_*C*H_2_CH_2_-), 22.6 (-(CH_2_)_4_*C*H_2_CH_3_), 14.1 (-(CH_2_)_5_*C*H_3_) ppm.

### S1.2. Refinement

H atoms were positioned with idealized geometry and refined using a riding model with *U*_iso_(H) = 1.2 · *U*_eq_(C) (1.5 for methyl H atoms). In one of the two crystallographically independent molecules the C atoms of one alkyl chain (C17—C19) and in the second molecule the ring C atoms C34—C35 are disordered over two positions and were refined using a split model. The site occupation factors were set to 0.65:0.35 for C17—C19 and C17'-C19', respectively, and to 0.70:0.30 for C34, C35 and C34', C35', respectively. The bonds C17—C18 and C18—C19 as well as the bond angles at C18 and C18' were restrained to the same value using the SAME instruction in SHELXL.

### Crystal data

**Table:**

C_28_H_36_I_2_S_3_	*V* = 2915.60 (16) Å^3^
*M**_r_* = 722.55	*Z* = 4
Triclinic, *P*1	*F*(000) = 1432
Hall symbol: -P 1	*D*_x_ = 1.646 Mg m^−^^3^
*a* = 13.4491 (4) Å	Mo *K*α radiation, λ = 0.71073 Å
*b* = 14.9488 (5) Å	θ = 1.4–27.0°
*c* = 16.1260 (5) Å	µ = 2.39 mm^−^^1^
α = 73.387 (2)°	*T* = 200 K
β = 71.208 (2)°	Block, yellow
γ = 77.794 (3)°	0.16 × 0.10 × 0.08 mm

### Data collection

**Table:**

Stoe IPDS-1 diffractometer	12585 independent reflections
Radiation source: fine-focus sealed tube	9932 reflections with *I* > 2σ(*I*)
Graphite monochromator	*R*_int_ = 0.038
ω scans	θ_max_ = 27.0°, θ_min_ = 1.4°
Absorption correction: numerical (*X-SHAPE* and *X-RED32*; Stoe & Cie, 2008)	*h* = −17→13
*T*_min_ = 0.748, *T*_max_ = 0.815	*k* = −17→19
26585 measured reflections	*l* = −20→20

### Refinement

**Table:**

Refinement on *F*^2^	Primary atom site location: structure-invariant direct methods
Least-squares matrix: full	Secondary atom site location: difference Fourier map
*R*[*F*^2^ > 2σ(*F*^2^)] = 0.034	Hydrogen site location: inferred from neighbouring sites
*wR*(*F*^2^) = 0.085	H-atom parameters constrained
*S* = 0.98	*w* = 1/[σ^2^(*F*_o_^2^) + (0.0524*P*)^2^] where *P* = (*F*_o_^2^ + 2*F*_c_^2^)/3
12585 reflections	(Δ/σ)_max_ = 0.002
640 parameters	Δρ_max_ = 1.01 e Å^−^^3^
3 restraints	Δρ_min_ = −0.81 e Å^−^^3^

### Special details

**Table:**

Geometry. All e.s.d.'s (except the e.s.d. in the dihedral angle between two l.s. planes) are estimated using the full covariance matrix. The cell e.s.d.'s are taken into account individually in the estimation of e.s.d.'s in distances, angles and torsion angles; correlations between e.s.d.'s in cell parameters are only used when they are defined by crystal symmetry. An approximate (isotropic) treatment of cell e.s.d.'s is used for estimating e.s.d.'s involving l.s. planes.
Refinement. Refinement of *F*^2^ against ALL reflections. The weighted *R*-factor *wR* and goodness of fit *S* are based on *F*^2^, conventional *R*-factors *R* are based on *F*, with *F* set to zero for negative *F*^2^. The threshold expression of *F*^2^ > σ(*F*^2^) is used only for calculating *R*-factors(gt) *etc*. and is not relevant to the choice of reflections for refinement. *R*-factors based on *F*^2^ are statistically about twice as large as those based on *F*, and *R*- factors based on ALL data will be even larger.

### Fractional atomic coordinates and isotropic or equivalent isotropic displacement parameters (Å^2^)

**Table:**

	*x*	*y*	*z*	*U*_iso_*/*U*_eq_	Occ. (<1)
S1	0.45567 (6)	0.37865 (6)	0.67496 (5)	0.03750 (16)	
C1	0.4026 (2)	0.4189 (2)	0.77285 (19)	0.0365 (6)	
C2	0.4555 (3)	0.4893 (2)	0.7707 (2)	0.0369 (6)	
C3	0.4291 (3)	0.5402 (3)	0.8449 (2)	0.0460 (8)	
H3A	0.4175	0.4937	0.9038	0.055*	
H3B	0.3624	0.5836	0.8447	0.055*	
C4	0.5162 (4)	0.5958 (3)	0.8346 (3)	0.0611 (10)	
H4A	0.4889	0.6400	0.8748	0.073*	
H4B	0.5758	0.5521	0.8532	0.073*	
C5	0.5555 (4)	0.6499 (3)	0.7399 (3)	0.0579 (10)	
H5A	0.6088	0.6884	0.7368	0.069*	
H5B	0.4957	0.6934	0.7215	0.069*	
C6	0.6050 (3)	0.5866 (2)	0.6743 (2)	0.0439 (7)	
H6A	0.6142	0.6253	0.6119	0.053*	
H6B	0.6761	0.5568	0.6809	0.053*	
C7	0.5385 (3)	0.5110 (2)	0.6899 (2)	0.0363 (6)	
C8	0.5497 (3)	0.4561 (2)	0.6313 (2)	0.0366 (6)	
C11	0.3116 (2)	0.3797 (2)	0.83951 (19)	0.0373 (7)	
S2	0.23062 (7)	0.43471 (7)	0.92356 (6)	0.0461 (2)	
C12	0.1472 (3)	0.3502 (3)	0.9634 (2)	0.0434 (7)	
I1	0.02175 (2)	0.35889 (2)	1.077875 (16)	0.05806 (8)	
C13	0.1778 (3)	0.2817 (2)	0.9163 (2)	0.0415 (7)	
C14	0.2721 (3)	0.3003 (2)	0.8453 (2)	0.0405 (7)	
H14	0.3052	0.2604	0.8048	0.049*	
C15	0.1216 (3)	0.1987 (3)	0.9326 (2)	0.0500 (8)	
H15A	0.1750	0.1441	0.9197	0.060*	
H15B	0.0815	0.1823	0.9971	0.060*	
C16	0.0469 (4)	0.2163 (3)	0.8766 (3)	0.0669 (12)	
H16A	0.0798	0.2494	0.8139	0.080*	0.65
H16B	−0.0191	0.2556	0.9015	0.080*	0.65
H16C	0.0920	0.2255	0.8134	0.080*	0.35
H16D	0.0052	0.2783	0.8825	0.080*	0.35
C17	0.0222 (6)	0.1174 (5)	0.8787 (4)	0.0491 (14)	0.65
H17A	0.0888	0.0783	0.8545	0.059*	0.65
H17B	−0.0102	0.0847	0.9416	0.059*	0.65
C18	−0.0533 (7)	0.1300 (6)	0.8222 (5)	0.0706 (19)	0.65
H18A	−0.1205	0.1667	0.8491	0.085*	0.65
H18B	−0.0222	0.1676	0.7611	0.085*	0.65
C19	−0.0785 (11)	0.0394 (9)	0.8134 (13)	0.086 (4)	0.65
H19A	−0.0944	−0.0035	0.8741	0.103*	0.65
H19B	−0.1440	0.0546	0.7939	0.103*	0.65
C17'	−0.0328 (12)	0.1541 (10)	0.8842 (8)	0.057 (3)	0.35
H17C	−0.0524	0.1129	0.9455	0.069*	0.35
H17D	−0.0978	0.1928	0.8709	0.069*	0.35
C18'	0.0222 (10)	0.0962 (9)	0.8153 (8)	0.055 (3)	0.35
H18C	0.0930	0.0684	0.8234	0.066*	0.35
H18D	0.0331	0.1391	0.7545	0.066*	0.35
C19'	−0.0350 (16)	0.0173 (14)	0.8183 (19)	0.068 (5)	0.35
H19C	−0.0310	−0.0341	0.8722	0.082*	0.35
H19D	−0.1107	0.0413	0.8234	0.082*	0.35
C20	0.0046 (8)	−0.0140 (7)	0.7498 (5)	0.137 (3)	
H20A	−0.0228	−0.0692	0.7486	0.206*	0.65
H20B	0.0686	−0.0345	0.7705	0.206*	0.65
H20C	0.0220	0.0271	0.6891	0.206*	0.65
H20D	−0.0285	−0.0689	0.7562	0.206*	0.35
H20E	0.0809	−0.0325	0.7416	0.206*	0.35
H20F	−0.0073	0.0348	0.6973	0.206*	0.35
C21	0.6228 (3)	0.4527 (2)	0.5433 (2)	0.0369 (6)	
S3	0.72642 (7)	0.52062 (6)	0.49196 (5)	0.04087 (17)	
C22	0.7682 (3)	0.4704 (2)	0.4008 (2)	0.0403 (7)	
I2	0.898985 (19)	0.513595 (18)	0.295482 (14)	0.04890 (7)	
C23	0.7078 (2)	0.4054 (2)	0.4071 (2)	0.0373 (7)	
C24	0.6247 (3)	0.3963 (2)	0.4889 (2)	0.0389 (7)	
H24	0.5740	0.3542	0.5048	0.047*	
C25	0.7271 (3)	0.3473 (3)	0.3395 (2)	0.0431 (7)	
H25A	0.7538	0.3863	0.2781	0.052*	
H25B	0.6590	0.3286	0.3434	0.052*	
C26	0.8066 (3)	0.2587 (3)	0.3544 (2)	0.0486 (8)	
H26A	0.8123	0.2219	0.3105	0.058*	
H26B	0.8771	0.2777	0.3421	0.058*	
C27	0.7771 (3)	0.1957 (3)	0.4494 (3)	0.0531 (9)	
H27A	0.7782	0.2307	0.4928	0.064*	
H27B	0.7038	0.1819	0.4638	0.064*	
C28	0.8498 (4)	0.1031 (3)	0.4625 (3)	0.0736 (13)	
H28A	0.9243	0.1160	0.4369	0.088*	
H28B	0.8381	0.0627	0.4284	0.088*	
C29	0.8330 (6)	0.0489 (5)	0.5621 (5)	0.123 (3)	
H29A	0.8822	−0.0106	0.5642	0.147*	
H29B	0.8531	0.0868	0.5940	0.147*	
C30	0.7303 (7)	0.0271 (6)	0.6098 (5)	0.143 (3)	
H30A	0.7275	−0.0028	0.6729	0.215*	
H30B	0.7118	−0.0162	0.5831	0.215*	
H30C	0.6799	0.0850	0.6065	0.215*	
S4	0.47208 (7)	0.86002 (6)	0.18105 (5)	0.03916 (17)	
C31	0.4655 (3)	0.7968 (2)	0.1083 (2)	0.0383 (7)	
C32	0.5396 (3)	0.8201 (2)	0.0258 (2)	0.0393 (7)	
C33	0.5569 (3)	0.7771 (3)	−0.0525 (2)	0.0505 (9)	
H33A	0.4875	0.7744	−0.0602	0.061*	0.70
H33B	0.5933	0.7119	−0.0398	0.061*	0.70
H33C	0.5440	0.7105	−0.0298	0.061*	0.30
H33D	0.5064	0.8111	−0.0877	0.061*	0.30
C34	0.6264 (4)	0.8375 (5)	−0.1429 (3)	0.0486 (12)	0.70
H34A	0.6490	0.8022	−0.1907	0.058*	0.70
H34B	0.5835	0.8974	−0.1635	0.058*	0.70
C35	0.7216 (6)	0.8577 (5)	−0.1263 (4)	0.0481 (15)	0.70
H35A	0.7694	0.8879	−0.1840	0.058*	0.70
H35B	0.7608	0.7981	−0.1001	0.058*	0.70
C34'	0.6651 (10)	0.7826 (9)	−0.1100 (8)	0.043 (2)	0.30
H34C	0.6772	0.7555	−0.1622	0.052*	0.30
H34D	0.7165	0.7477	−0.0758	0.052*	0.30
C35'	0.6773 (15)	0.8860 (12)	−0.1410 (8)	0.048 (4)	0.30
H35C	0.6153	0.9220	−0.1613	0.058*	0.30
H35D	0.7415	0.8958	−0.1926	0.058*	0.30
C36	0.6868 (3)	0.9229 (3)	−0.0616 (2)	0.0454 (8)	
H36A	0.7487	0.9282	−0.0435	0.054*	0.70
H36B	0.6612	0.9866	−0.0932	0.054*	0.70
H36C	0.7571	0.8989	−0.0508	0.054*	0.30
H36D	0.6788	0.9926	−0.0774	0.054*	0.30
C37	0.6008 (3)	0.8884 (2)	0.0213 (2)	0.0383 (7)	
C38	0.5744 (3)	0.9171 (2)	0.1006 (2)	0.0378 (7)	
C41	0.3850 (3)	0.7341 (2)	0.1398 (2)	0.0388 (7)	
S5	0.37041 (8)	0.66872 (7)	0.07245 (5)	0.0473 (2)	
C42	0.2639 (3)	0.6232 (2)	0.1546 (2)	0.0426 (7)	
I3	0.18641 (2)	0.532520 (19)	0.128836 (16)	0.05364 (7)	
C43	0.2389 (3)	0.6558 (2)	0.2308 (2)	0.0409 (7)	
C44	0.3098 (3)	0.7179 (2)	0.2210 (2)	0.0433 (7)	
H44	0.3055	0.7465	0.2678	0.052*	
C45	0.1470 (3)	0.6270 (3)	0.3120 (2)	0.0509 (9)	
H45A	0.1654	0.5605	0.3417	0.061*	
H45B	0.0849	0.6301	0.2905	0.061*	
C46	0.1148 (3)	0.6850 (3)	0.3818 (2)	0.0460 (8)	
H46A	0.1012	0.7524	0.3522	0.055*	
H46B	0.1736	0.6771	0.4087	0.055*	
C47	0.0160 (3)	0.6556 (3)	0.4559 (2)	0.0503 (8)	
H47A	−0.0417	0.6616	0.4282	0.060*	
H47B	0.0307	0.5883	0.4856	0.060*	
C48	−0.0217 (3)	0.7121 (3)	0.5265 (3)	0.0561 (9)	
H48A	0.0366	0.7071	0.5532	0.067*	
H48B	−0.0375	0.7791	0.4968	0.067*	
C49	−0.1183 (3)	0.6826 (3)	0.6012 (3)	0.0612 (10)	
H49A	−0.1048	0.6144	0.6279	0.073*	
H49B	−0.1785	0.6929	0.5754	0.073*	
C50	−0.1489 (5)	0.7351 (5)	0.6747 (4)	0.0949 (18)	
H50A	−0.2117	0.7124	0.7213	0.142*	
H50B	−0.1646	0.8026	0.6492	0.142*	
H50C	−0.0902	0.7244	0.7014	0.142*	
C51	0.6176 (3)	0.9826 (2)	0.1260 (2)	0.0375 (7)	
S6	0.70220 (7)	1.06090 (6)	0.04923 (5)	0.04254 (18)	
C52	0.7062 (3)	1.1091 (2)	0.1330 (2)	0.0402 (7)	
I4	0.79965 (2)	1.214701 (18)	0.100870 (15)	0.05306 (7)	
C53	0.6466 (3)	1.0684 (2)	0.2165 (2)	0.0401 (7)	
C54	0.5969 (3)	0.9964 (2)	0.2108 (2)	0.0427 (7)	
H54	0.5523	0.9602	0.2622	0.051*	
C55	0.6328 (3)	1.0955 (3)	0.3029 (2)	0.0470 (8)	
H55A	0.6564	1.1579	0.2883	0.056*	
H55B	0.6787	1.0493	0.3369	0.056*	
C56	0.5186 (3)	1.0994 (3)	0.3625 (2)	0.0439 (7)	
H56A	0.4711	1.1379	0.3259	0.053*	
H56B	0.4985	1.0349	0.3850	0.053*	
C57	0.5026 (3)	1.1411 (3)	0.4426 (2)	0.0461 (8)	
H57A	0.5473	1.1005	0.4808	0.055*	
H57B	0.5269	1.2040	0.4199	0.055*	
C58	0.3886 (3)	1.1508 (3)	0.4999 (2)	0.0515 (9)	
H58A	0.3666	1.0872	0.5274	0.062*	
H58B	0.3429	1.1860	0.4605	0.062*	
C59	0.3710 (4)	1.2009 (3)	0.5741 (2)	0.0610 (11)	
H59A	0.4191	1.1674	0.6117	0.073*	
H59B	0.3896	1.2656	0.5464	0.073*	
C60	0.2579 (5)	1.2063 (4)	0.6339 (3)	0.0900 (17)	
H60A	0.2514	1.2390	0.6805	0.135*	
H60B	0.2393	1.1425	0.6625	0.135*	
H60C	0.2099	1.2409	0.5974	0.135*	

### Atomic displacement parameters (Å^2^)

**Table:**

	*U*^11^	*U*^22^	*U*^33^	*U*^12^	*U*^13^	*U*^23^
S1	0.0356 (4)	0.0392 (4)	0.0378 (3)	−0.0099 (3)	−0.0046 (3)	−0.0120 (3)
C1	0.0327 (15)	0.0405 (17)	0.0362 (14)	−0.0038 (13)	−0.0078 (12)	−0.0115 (12)
C2	0.0337 (16)	0.0366 (17)	0.0408 (15)	−0.0012 (13)	−0.0110 (13)	−0.0118 (12)
C3	0.0455 (19)	0.051 (2)	0.0446 (16)	−0.0092 (16)	−0.0082 (14)	−0.0196 (15)
C4	0.064 (3)	0.068 (3)	0.062 (2)	−0.020 (2)	−0.0126 (19)	−0.0288 (19)
C5	0.072 (3)	0.049 (2)	0.059 (2)	−0.024 (2)	−0.0078 (19)	−0.0210 (17)
C6	0.0444 (19)	0.0410 (18)	0.0492 (17)	−0.0127 (15)	−0.0097 (15)	−0.0136 (14)
C7	0.0356 (16)	0.0310 (15)	0.0423 (15)	−0.0043 (12)	−0.0117 (13)	−0.0080 (12)
C8	0.0345 (16)	0.0345 (16)	0.0390 (14)	−0.0045 (13)	−0.0082 (12)	−0.0083 (12)
C11	0.0311 (15)	0.0435 (18)	0.0362 (14)	−0.0038 (13)	−0.0074 (12)	−0.0109 (12)
S2	0.0412 (4)	0.0539 (5)	0.0443 (4)	−0.0139 (4)	0.0001 (3)	−0.0215 (4)
C12	0.0317 (16)	0.055 (2)	0.0403 (15)	−0.0106 (15)	−0.0010 (13)	−0.0137 (14)
I1	0.04650 (14)	0.07347 (18)	0.04928 (13)	−0.01999 (12)	0.00718 (10)	−0.02162 (11)
C13	0.0359 (17)	0.0438 (18)	0.0408 (15)	−0.0079 (14)	−0.0075 (13)	−0.0055 (13)
C14	0.0373 (17)	0.0398 (18)	0.0419 (15)	−0.0058 (14)	−0.0069 (13)	−0.0099 (13)
C15	0.049 (2)	0.0397 (19)	0.0555 (19)	−0.0131 (16)	−0.0043 (16)	−0.0091 (15)
C16	0.085 (3)	0.074 (3)	0.0485 (19)	−0.047 (3)	−0.012 (2)	−0.0058 (18)
C17	0.058 (4)	0.041 (4)	0.049 (3)	−0.013 (3)	−0.010 (3)	−0.012 (3)
C18	0.076 (5)	0.076 (5)	0.074 (4)	−0.025 (4)	−0.033 (4)	−0.014 (4)
C19	0.081 (9)	0.102 (9)	0.088 (7)	−0.046 (7)	−0.012 (7)	−0.033 (7)
C17'	0.056 (8)	0.059 (9)	0.057 (6)	−0.020 (6)	−0.002 (6)	−0.020 (6)
C18'	0.048 (7)	0.061 (7)	0.058 (6)	−0.026 (6)	−0.006 (5)	−0.013 (5)
C19'	0.069 (14)	0.078 (11)	0.065 (9)	−0.031 (10)	−0.010 (11)	−0.021 (8)
C20	0.153 (8)	0.152 (8)	0.123 (6)	−0.028 (6)	−0.032 (5)	−0.059 (5)
C21	0.0330 (16)	0.0351 (16)	0.0412 (15)	−0.0048 (13)	−0.0083 (13)	−0.0089 (12)
S3	0.0392 (4)	0.0409 (4)	0.0423 (4)	−0.0131 (3)	−0.0045 (3)	−0.0116 (3)
C22	0.0355 (16)	0.0444 (18)	0.0383 (14)	−0.0072 (14)	−0.0049 (13)	−0.0105 (13)
I2	0.04303 (13)	0.05674 (15)	0.04192 (11)	−0.01741 (10)	−0.00180 (9)	−0.00759 (9)
C23	0.0314 (15)	0.0404 (17)	0.0378 (14)	−0.0035 (13)	−0.0093 (12)	−0.0072 (12)
C24	0.0354 (16)	0.0386 (17)	0.0433 (15)	−0.0091 (13)	−0.0094 (13)	−0.0093 (13)
C25	0.0442 (18)	0.050 (2)	0.0390 (15)	−0.0101 (15)	−0.0124 (14)	−0.0122 (13)
C26	0.046 (2)	0.056 (2)	0.0514 (18)	−0.0076 (16)	−0.0116 (15)	−0.0254 (16)
C27	0.051 (2)	0.045 (2)	0.066 (2)	−0.0058 (17)	−0.0192 (18)	−0.0142 (16)
C28	0.071 (3)	0.050 (3)	0.093 (3)	0.002 (2)	−0.018 (3)	−0.019 (2)
C29	0.095 (5)	0.077 (4)	0.156 (6)	0.006 (4)	−0.037 (5)	0.023 (4)
C30	0.120 (7)	0.122 (6)	0.127 (6)	0.003 (5)	−0.015 (5)	0.027 (5)
S4	0.0433 (4)	0.0374 (4)	0.0381 (3)	−0.0120 (3)	−0.0072 (3)	−0.0108 (3)
C31	0.0379 (17)	0.0375 (17)	0.0411 (15)	−0.0051 (13)	−0.0108 (13)	−0.0120 (12)
C32	0.0401 (17)	0.0372 (17)	0.0431 (15)	−0.0022 (14)	−0.0129 (13)	−0.0142 (13)
C33	0.047 (2)	0.061 (2)	0.0483 (18)	−0.0110 (17)	−0.0076 (15)	−0.0240 (16)
C34	0.043 (3)	0.059 (4)	0.045 (3)	−0.008 (3)	−0.005 (2)	−0.020 (3)
C35	0.047 (4)	0.054 (4)	0.042 (3)	−0.008 (3)	−0.007 (3)	−0.013 (3)
C34'	0.042 (6)	0.045 (7)	0.039 (5)	0.000 (5)	−0.002 (5)	−0.018 (5)
C35'	0.060 (10)	0.060 (10)	0.023 (5)	−0.014 (8)	−0.007 (6)	−0.008 (5)
C36	0.0458 (19)	0.050 (2)	0.0383 (15)	−0.0099 (16)	−0.0077 (14)	−0.0099 (14)
C37	0.0379 (17)	0.0366 (17)	0.0390 (14)	−0.0036 (13)	−0.0124 (13)	−0.0061 (12)
C38	0.0380 (17)	0.0331 (16)	0.0381 (14)	−0.0053 (13)	−0.0065 (12)	−0.0062 (12)
C41	0.0401 (17)	0.0370 (17)	0.0419 (15)	−0.0043 (14)	−0.0124 (13)	−0.0128 (12)
S5	0.0564 (5)	0.0510 (5)	0.0397 (4)	−0.0211 (4)	−0.0071 (4)	−0.0155 (3)
C42	0.0479 (19)	0.0400 (18)	0.0457 (16)	−0.0119 (15)	−0.0141 (14)	−0.0132 (13)
I3	0.05975 (16)	0.06070 (16)	0.05085 (12)	−0.02589 (12)	−0.01073 (11)	−0.02133 (10)
C43	0.0407 (18)	0.0458 (19)	0.0411 (15)	−0.0101 (14)	−0.0120 (13)	−0.0138 (13)
C44	0.0449 (19)	0.0458 (19)	0.0453 (16)	−0.0091 (15)	−0.0120 (14)	−0.0186 (14)
C45	0.046 (2)	0.057 (2)	0.0526 (19)	−0.0167 (17)	−0.0035 (16)	−0.0226 (16)
C46	0.046 (2)	0.049 (2)	0.0450 (16)	−0.0104 (16)	−0.0076 (15)	−0.0159 (14)
C47	0.047 (2)	0.059 (2)	0.0487 (18)	−0.0158 (17)	−0.0071 (15)	−0.0182 (16)
C48	0.051 (2)	0.065 (3)	0.0522 (19)	−0.0120 (19)	−0.0051 (17)	−0.0212 (17)
C49	0.047 (2)	0.076 (3)	0.056 (2)	−0.009 (2)	−0.0033 (17)	−0.0207 (19)
C50	0.077 (4)	0.123 (5)	0.078 (3)	−0.014 (3)	0.016 (3)	−0.055 (3)
C51	0.0388 (17)	0.0336 (16)	0.0377 (14)	−0.0060 (13)	−0.0094 (13)	−0.0053 (12)
S6	0.0466 (5)	0.0427 (4)	0.0374 (4)	−0.0157 (4)	−0.0043 (3)	−0.0095 (3)
C52	0.0400 (17)	0.0389 (17)	0.0439 (15)	−0.0120 (14)	−0.0100 (13)	−0.0101 (13)
I4	0.05746 (15)	0.05634 (15)	0.04892 (12)	−0.03000 (12)	−0.00790 (10)	−0.00943 (10)
C53	0.0409 (17)	0.0409 (18)	0.0384 (14)	−0.0110 (14)	−0.0087 (13)	−0.0079 (12)
C54	0.0454 (19)	0.0417 (18)	0.0397 (15)	−0.0152 (15)	−0.0076 (14)	−0.0056 (13)
C55	0.051 (2)	0.052 (2)	0.0406 (16)	−0.0184 (17)	−0.0108 (15)	−0.0089 (14)
C56	0.049 (2)	0.0407 (18)	0.0425 (16)	−0.0128 (15)	−0.0097 (14)	−0.0094 (13)
C57	0.054 (2)	0.045 (2)	0.0395 (15)	−0.0126 (16)	−0.0128 (15)	−0.0071 (13)
C58	0.060 (2)	0.047 (2)	0.0462 (17)	−0.0076 (17)	−0.0114 (16)	−0.0127 (15)
C59	0.076 (3)	0.061 (3)	0.0448 (18)	0.000 (2)	−0.0180 (18)	−0.0164 (17)
C60	0.086 (4)	0.105 (4)	0.064 (3)	0.012 (3)	−0.004 (3)	−0.036 (3)

### Geometric parameters (Å, º)

**Table:**

S1—C1	1.732 (3)	C30—H30B	0.9800
S1—C8	1.737 (3)	C30—H30C	0.9800
C1—C2	1.377 (5)	S4—C31	1.736 (3)
C1—C11	1.446 (4)	S4—C38	1.737 (3)
C2—C7	1.422 (4)	C31—C32	1.383 (4)
C2—C3	1.506 (4)	C31—C41	1.452 (5)
C3—C4	1.516 (5)	C32—C37	1.414 (5)
C3—H3A	0.9900	C32—C33	1.507 (4)
C3—H3B	0.9900	C33—C34'	1.457 (12)
C4—C5	1.491 (5)	C33—C34	1.594 (6)
C4—H4A	0.9900	C33—H33A	0.9900
C4—H4B	0.9900	C33—H33B	0.9900
C5—C6	1.520 (5)	C33—H33C	0.9900
C5—H5A	0.9900	C33—H33D	0.9900
C5—H5B	0.9900	C34—C35	1.494 (9)
C6—C7	1.504 (5)	C34—H34A	0.9900
C6—H6A	0.9900	C34—H34B	0.9900
C6—H6B	0.9900	C35—C36	1.527 (8)
C7—C8	1.374 (4)	C35—H35A	0.9900
C8—C21	1.451 (4)	C35—H35B	0.9900
C11—C14	1.368 (5)	C34'—C35'	1.51 (2)
C11—S2	1.735 (3)	C34'—H34C	0.9900
S2—C12	1.715 (4)	C34'—H34D	0.9900
C12—C13	1.364 (5)	C35'—C36	1.581 (15)
C12—I1	2.076 (3)	C35'—H35C	0.9900
C13—C14	1.424 (4)	C35'—H35D	0.9900
C13—C15	1.503 (5)	C36—C37	1.502 (4)
C14—H14	0.9500	C36—H36A	0.9900
C15—C16	1.494 (6)	C36—H36B	0.9900
C15—H15A	0.9900	C36—H36C	0.9900
C15—H15B	0.9900	C36—H36D	0.9900
C16—C17'	1.517 (13)	C37—C38	1.380 (4)
C16—C17	1.571 (8)	C38—C51	1.446 (5)
C16—H16A	0.9900	C41—C44	1.366 (5)
C16—H16B	0.9900	C41—S5	1.732 (3)
C16—H16C	0.9901	S5—C42	1.716 (4)
C16—H16D	0.9900	C42—C43	1.367 (4)
C17—C18	1.520 (8)	C42—I3	2.072 (3)
C17—H17A	0.9900	C43—C44	1.411 (5)
C17—H17B	0.9900	C43—C45	1.511 (5)
C18—C19	1.518 (11)	C44—H44	0.9500
C18—H18A	0.9900	C45—C46	1.514 (5)
C18—H18B	0.9900	C45—H45A	0.9900
C19—C20	1.513 (17)	C45—H45B	0.9900
C19—H19A	0.9900	C46—C47	1.520 (5)
C19—H19B	0.9900	C46—H46A	0.9900
C17'—C18'	1.510 (13)	C46—H46B	0.9900
C17'—H17C	0.9900	C47—C48	1.505 (5)
C17'—H17D	0.9900	C47—H47A	0.9900
C18'—C19'	1.521 (14)	C47—H47B	0.9900
C18'—H18C	0.9900	C48—C49	1.504 (5)
C18'—H18D	0.9900	C48—H48A	0.9900
C19'—C20	1.24 (3)	C48—H48B	0.9900
C19'—H19C	0.9900	C49—C50	1.507 (6)
C19'—H19D	0.9900	C49—H49A	0.9900
C20—H20A	0.9800	C49—H49B	0.9900
C20—H20B	0.9800	C50—H50A	0.9800
C20—H20C	0.9800	C50—H50B	0.9800
C20—H20D	0.9800	C50—H50C	0.9800
C20—H20E	0.9801	C51—C54	1.371 (4)
C20—H20F	0.9801	C51—S6	1.739 (3)
C21—C24	1.370 (4)	S6—C52	1.722 (3)
C21—S3	1.742 (3)	C52—C53	1.368 (4)
S3—C22	1.721 (3)	C52—I4	2.072 (3)
C22—C23	1.356 (5)	C53—C54	1.418 (5)
C22—I2	2.079 (3)	C53—C55	1.505 (4)
C23—C24	1.422 (4)	C54—H54	0.9500
C23—C25	1.508 (4)	C55—C56	1.527 (5)
C24—H24	0.9500	C55—H55A	0.9900
C25—C26	1.529 (5)	C55—H55B	0.9900
C25—H25A	0.9900	C56—C57	1.525 (5)
C25—H25B	0.9900	C56—H56A	0.9900
C26—C27	1.528 (5)	C56—H56B	0.9900
C26—H26A	0.9900	C57—C58	1.516 (5)
C26—H26B	0.9900	C57—H57A	0.9900
C27—C28	1.520 (6)	C57—H57B	0.9900
C27—H27A	0.9900	C58—C59	1.519 (5)
C27—H27B	0.9900	C58—H58A	0.9900
C28—C29	1.546 (8)	C58—H58B	0.9900
C28—H28A	0.9900	C59—C60	1.516 (7)
C28—H28B	0.9900	C59—H59A	0.9900
C29—C30	1.403 (10)	C59—H59B	0.9900
C29—H29A	0.9900	C60—H60A	0.9800
C29—H29B	0.9900	C60—H60B	0.9800
C30—H30A	0.9800	C60—H60C	0.9800
			
C1—S1—C8	92.38 (15)	C29—C30—H30A	109.5
C2—C1—C11	130.6 (3)	C29—C30—H30B	109.5
C2—C1—S1	110.7 (2)	H30A—C30—H30B	109.5
C11—C1—S1	118.6 (2)	C29—C30—H30C	109.5
C1—C2—C7	113.1 (3)	H30A—C30—H30C	109.5
C1—C2—C3	124.4 (3)	H30B—C30—H30C	109.5
C7—C2—C3	122.4 (3)	C31—S4—C38	92.22 (16)
C2—C3—C4	112.2 (3)	C32—C31—C41	131.2 (3)
C2—C3—H3A	109.2	C32—C31—S4	110.5 (2)
C4—C3—H3A	109.2	C41—C31—S4	118.2 (2)
C2—C3—H3B	109.2	C31—C32—C37	113.4 (3)
C4—C3—H3B	109.2	C31—C32—C33	124.6 (3)
H3A—C3—H3B	107.9	C37—C32—C33	122.1 (3)
C5—C4—C3	111.6 (3)	C34'—C33—C32	109.7 (5)
C5—C4—H4A	109.3	C32—C33—C34	110.6 (3)
C3—C4—H4A	109.3	C34'—C33—H33A	136.5
C5—C4—H4B	109.3	C32—C33—H33A	109.5
C3—C4—H4B	109.3	C34—C33—H33A	109.5
H4A—C4—H4B	108.0	C34'—C33—H33B	75.3
C4—C5—C6	112.6 (3)	C32—C33—H33B	109.5
C4—C5—H5A	109.1	C34—C33—H33B	109.5
C6—C5—H5A	109.1	H33A—C33—H33B	108.1
C4—C5—H5B	109.1	C34'—C33—H33C	109.7
C6—C5—H5B	109.1	C32—C33—H33C	109.7
H5A—C5—H5B	107.8	C34—C33—H33C	135.4
C7—C6—C5	112.1 (3)	H33A—C33—H33C	73.3
C7—C6—H6A	109.2	C34'—C33—H33D	109.7
C5—C6—H6A	109.2	C32—C33—H33D	109.7
C7—C6—H6B	109.2	C34—C33—H33D	74.8
C5—C6—H6B	109.2	H33B—C33—H33D	135.5
H6A—C6—H6B	107.9	H33C—C33—H33D	108.2
C8—C7—C2	113.1 (3)	C35—C34—C33	109.8 (5)
C8—C7—C6	125.7 (3)	C35—C34—H34A	109.7
C2—C7—C6	121.2 (3)	C33—C34—H34A	109.7
C7—C8—C21	132.2 (3)	C35—C34—H34B	109.7
C7—C8—S1	110.7 (2)	C33—C34—H34B	109.7
C21—C8—S1	117.2 (2)	H34A—C34—H34B	108.2
C14—C11—C1	128.0 (3)	C34—C35—C36	109.6 (5)
C14—C11—S2	109.5 (2)	C34—C35—H35A	109.8
C1—C11—S2	122.4 (2)	C36—C35—H35A	109.8
C12—S2—C11	91.84 (16)	C34—C35—H35B	109.8
C13—C12—S2	113.2 (2)	C36—C35—H35B	109.8
C13—C12—I1	128.6 (3)	H35A—C35—H35B	108.2
S2—C12—I1	118.06 (19)	C33—C34'—C35'	105.9 (11)
C12—C13—C14	110.2 (3)	C33—C34'—H34C	110.6
C12—C13—C15	126.7 (3)	C35'—C34'—H34C	110.6
C14—C13—C15	123.1 (3)	C33—C34'—H34D	110.6
C11—C14—C13	115.2 (3)	C35'—C34'—H34D	110.6
C11—C14—H14	122.4	H34C—C34'—H34D	108.7
C13—C14—H14	122.4	C34'—C35'—C36	110.4 (10)
C16—C15—C13	113.4 (3)	C34'—C35'—H35C	109.6
C16—C15—H15A	108.9	C36—C35'—H35C	109.6
C13—C15—H15A	108.9	C34'—C35'—H35D	109.6
C16—C15—H15B	108.9	C36—C35'—H35D	109.6
C13—C15—H15B	108.9	H35C—C35'—H35D	108.1
H15A—C15—H15B	107.7	C37—C36—C35	112.4 (4)
C15—C16—C17'	127.6 (7)	C37—C36—C35'	107.9 (6)
C15—C16—C17	106.9 (4)	C37—C36—H36A	109.1
C15—C16—H16A	110.3	C35—C36—H36A	109.1
C17'—C16—H16A	113.7	C35'—C36—H36A	132.2
C17—C16—H16A	110.3	C37—C36—H36B	109.1
C15—C16—H16B	110.3	C35—C36—H36B	109.1
C17'—C16—H16B	81.0	C35'—C36—H36B	87.1
C17—C16—H16B	110.3	H36A—C36—H36B	107.9
H16A—C16—H16B	108.6	C37—C36—H36C	110.1
C15—C16—H16C	105.5	C35—C36—H36C	84.4
C17'—C16—H16C	105.5	C35'—C36—H36C	110.1
C17—C16—H16C	93.3	H36B—C36—H36C	128.9
H16B—C16—H16C	127.9	C37—C36—H36D	110.1
C15—C16—H16D	105.4	C35—C36—H36D	127.4
C17'—C16—H16D	105.3	C35'—C36—H36D	110.1
C17—C16—H16D	136.0	H36C—C36—H36D	108.4
H16A—C16—H16D	85.1	C38—C37—C32	113.1 (3)
H16C—C16—H16D	106.0	C38—C37—C36	124.7 (3)
C18—C17—C16	109.7 (5)	C32—C37—C36	122.3 (3)
C18—C17—H17A	109.7	C37—C38—C51	131.4 (3)
C16—C17—H17A	109.7	C37—C38—S4	110.8 (3)
C18—C17—H17B	109.7	C51—C38—S4	117.8 (2)
C16—C17—H17B	109.7	C44—C41—C31	128.5 (3)
H17A—C17—H17B	108.2	C44—C41—S5	109.4 (2)
C19—C18—C17	115.3 (8)	C31—C41—S5	122.1 (2)
C19—C18—H18A	108.4	C42—S5—C41	91.75 (16)
C17—C18—H18A	108.4	C43—C42—S5	113.0 (3)
C19—C18—H18B	108.4	C43—C42—I3	127.7 (3)
C17—C18—H18B	108.4	S5—C42—I3	119.30 (17)
H18A—C18—H18B	107.5	C42—C43—C44	110.3 (3)
C20—C19—C18	117.9 (11)	C42—C43—C45	122.8 (3)
C20—C19—H19A	107.8	C44—C43—C45	126.9 (3)
C18—C19—H19A	107.8	C41—C44—C43	115.6 (3)
C20—C19—H19B	107.8	C41—C44—H44	122.2
C18—C19—H19B	107.8	C43—C44—H44	122.2
H19A—C19—H19B	107.2	C43—C45—C46	116.0 (3)
C18'—C17'—C16	105.3 (9)	C43—C45—H45A	108.3
C18'—C17'—H17C	110.7	C46—C45—H45A	108.3
C16—C17'—H17C	110.7	C43—C45—H45B	108.3
C18'—C17'—H17D	110.7	C46—C45—H45B	108.3
C16—C17'—H17D	110.7	H45A—C45—H45B	107.4
H17C—C17'—H17D	108.8	C45—C46—C47	111.5 (3)
C17'—C18'—C19'	115.9 (12)	C45—C46—H46A	109.3
C17'—C18'—H18C	108.3	C47—C46—H46A	109.3
C19'—C18'—H18C	108.3	C45—C46—H46B	109.3
C17'—C18'—H18D	108.3	C47—C46—H46B	109.3
C19'—C18'—H18D	108.3	H46A—C46—H46B	108.0
H18C—C18'—H18D	107.4	C48—C47—C46	114.1 (3)
C20—C19'—C18'	111.1 (17)	C48—C47—H47A	108.7
C20—C19'—H19C	109.4	C46—C47—H47A	108.7
C18'—C19'—H19C	109.4	C48—C47—H47B	108.7
C20—C19'—H19D	109.4	C46—C47—H47B	108.7
C18'—C19'—H19D	109.4	H47A—C47—H47B	107.6
H19C—C19'—H19D	108.0	C49—C48—C47	114.7 (4)
C19'—C20—H20A	116.7	C49—C48—H48A	108.6
C19—C20—H20A	109.5	C47—C48—H48A	108.6
C19'—C20—H20B	86.0	C49—C48—H48B	108.6
C19—C20—H20B	109.5	C47—C48—H48B	108.6
H20A—C20—H20B	109.5	H48A—C48—H48B	107.6
C19'—C20—H20C	122.4	C48—C49—C50	113.5 (4)
C19—C20—H20C	109.5	C48—C49—H49A	108.9
H20A—C20—H20C	109.5	C50—C49—H49A	108.9
H20B—C20—H20C	109.5	C48—C49—H49B	108.9
C19'—C20—H20D	109.5	C50—C49—H49B	108.9
C19—C20—H20D	103.0	H49A—C49—H49B	107.7
H20B—C20—H20D	109.8	C49—C50—H50A	109.5
H20C—C20—H20D	115.4	C49—C50—H50B	109.5
C19'—C20—H20E	109.6	H50A—C50—H50B	109.5
C19—C20—H20E	131.8	C49—C50—H50C	109.5
H20A—C20—H20E	105.9	H50A—C50—H50C	109.5
H20C—C20—H20E	87.7	H50B—C50—H50C	109.5
H20D—C20—H20E	109.5	C54—C51—C38	127.0 (3)
C19'—C20—H20F	109.3	C54—C51—S6	109.4 (2)
C19—C20—H20F	91.3	C38—C51—S6	123.5 (2)
H20A—C20—H20F	105.6	C52—S6—C51	91.69 (15)
H20B—C20—H20F	129.4	C53—C52—S6	113.1 (2)
H20D—C20—H20F	109.5	C53—C52—I4	127.3 (2)
H20E—C20—H20F	109.5	S6—C52—I4	119.49 (17)
C24—C21—C8	127.2 (3)	C52—C53—C54	110.2 (3)
C24—C21—S3	109.4 (2)	C52—C53—C55	125.9 (3)
C8—C21—S3	123.4 (2)	C54—C53—C55	123.9 (3)
C22—S3—C21	91.49 (16)	C51—C54—C53	115.6 (3)
C23—C22—S3	113.4 (2)	C51—C54—H54	122.2
C23—C22—I2	127.3 (2)	C53—C54—H54	122.2
S3—C22—I2	119.31 (18)	C53—C55—C56	113.1 (3)
C22—C23—C24	110.5 (3)	C53—C55—H55A	109.0
C22—C23—C25	125.6 (3)	C56—C55—H55A	109.0
C24—C23—C25	123.9 (3)	C53—C55—H55B	109.0
C21—C24—C23	115.2 (3)	C56—C55—H55B	109.0
C21—C24—H24	122.4	H55A—C55—H55B	107.8
C23—C24—H24	122.4	C57—C56—C55	112.4 (3)
C23—C25—C26	112.8 (3)	C57—C56—H56A	109.1
C23—C25—H25A	109.0	C55—C56—H56A	109.1
C26—C25—H25A	109.0	C57—C56—H56B	109.1
C23—C25—H25B	109.0	C55—C56—H56B	109.1
C26—C25—H25B	109.0	H56A—C56—H56B	107.8
H25A—C25—H25B	107.8	C58—C57—C56	113.4 (3)
C27—C26—C25	113.8 (3)	C58—C57—H57A	108.9
C27—C26—H26A	108.8	C56—C57—H57A	108.9
C25—C26—H26A	108.8	C58—C57—H57B	108.9
C27—C26—H26B	108.8	C56—C57—H57B	108.9
C25—C26—H26B	108.8	H57A—C57—H57B	107.7
H26A—C26—H26B	107.7	C57—C58—C59	113.4 (3)
C28—C27—C26	114.2 (3)	C57—C58—H58A	108.9
C28—C27—H27A	108.7	C59—C58—H58A	108.9
C26—C27—H27A	108.7	C57—C58—H58B	108.9
C28—C27—H27B	108.7	C59—C58—H58B	108.9
C26—C27—H27B	108.7	H58A—C58—H58B	107.7
H27A—C27—H27B	107.6	C60—C59—C58	113.1 (4)
C27—C28—C29	113.7 (4)	C60—C59—H59A	109.0
C27—C28—H28A	108.8	C58—C59—H59A	109.0
C29—C28—H28A	108.8	C60—C59—H59B	109.0
C27—C28—H28B	108.8	C58—C59—H59B	109.0
C29—C28—H28B	108.8	H59A—C59—H59B	107.8
H28A—C28—H28B	107.7	C59—C60—H60A	109.5
C30—C29—C28	116.4 (7)	C59—C60—H60B	109.5
C30—C29—H29A	108.2	H60A—C60—H60B	109.5
C28—C29—H29A	108.2	C59—C60—H60C	109.5
C30—C29—H29B	108.2	H60A—C60—H60C	109.5
C28—C29—H29B	108.2	H60B—C60—H60C	109.5
H29A—C29—H29B	107.3		
