# Supplementary figures and images for: Crystal structure of 1,3-bis­(4-hexyl-5-iodo­thio­phen-2-yl)-4,5,6,7-tetra­hydro-2-benzo­thio­phene
Source: Acta Crystallogr Sect E Struct Rep Online. 2014 Sep 27;70(Pt 10):o1133–4. doi: 10.1107/S1600536814019667 (PMC4257205; doi:10.1107/S1600536814019667)

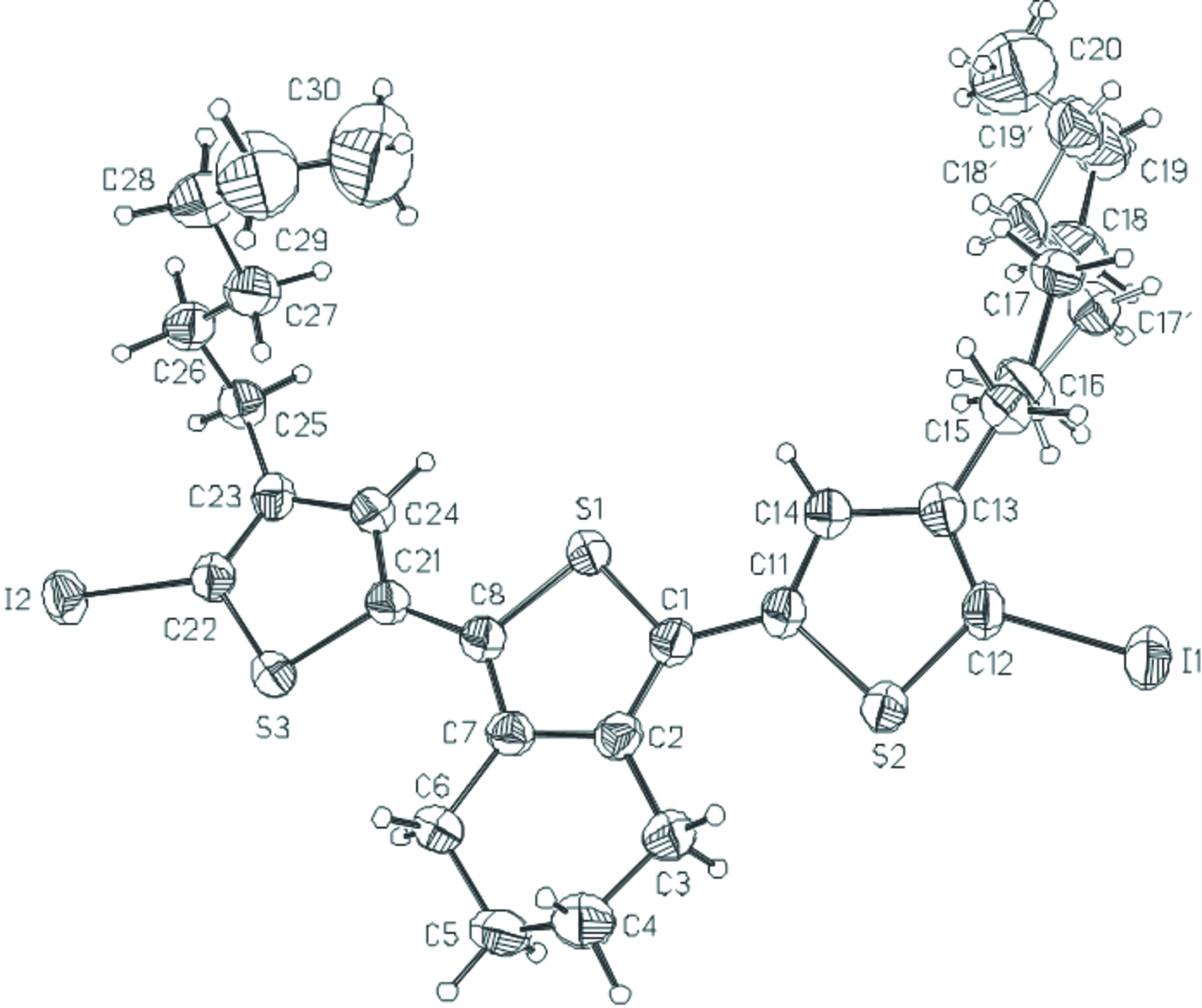

Supplement: Supplementary file 4 [file e-70-o1133-fig1.tif]

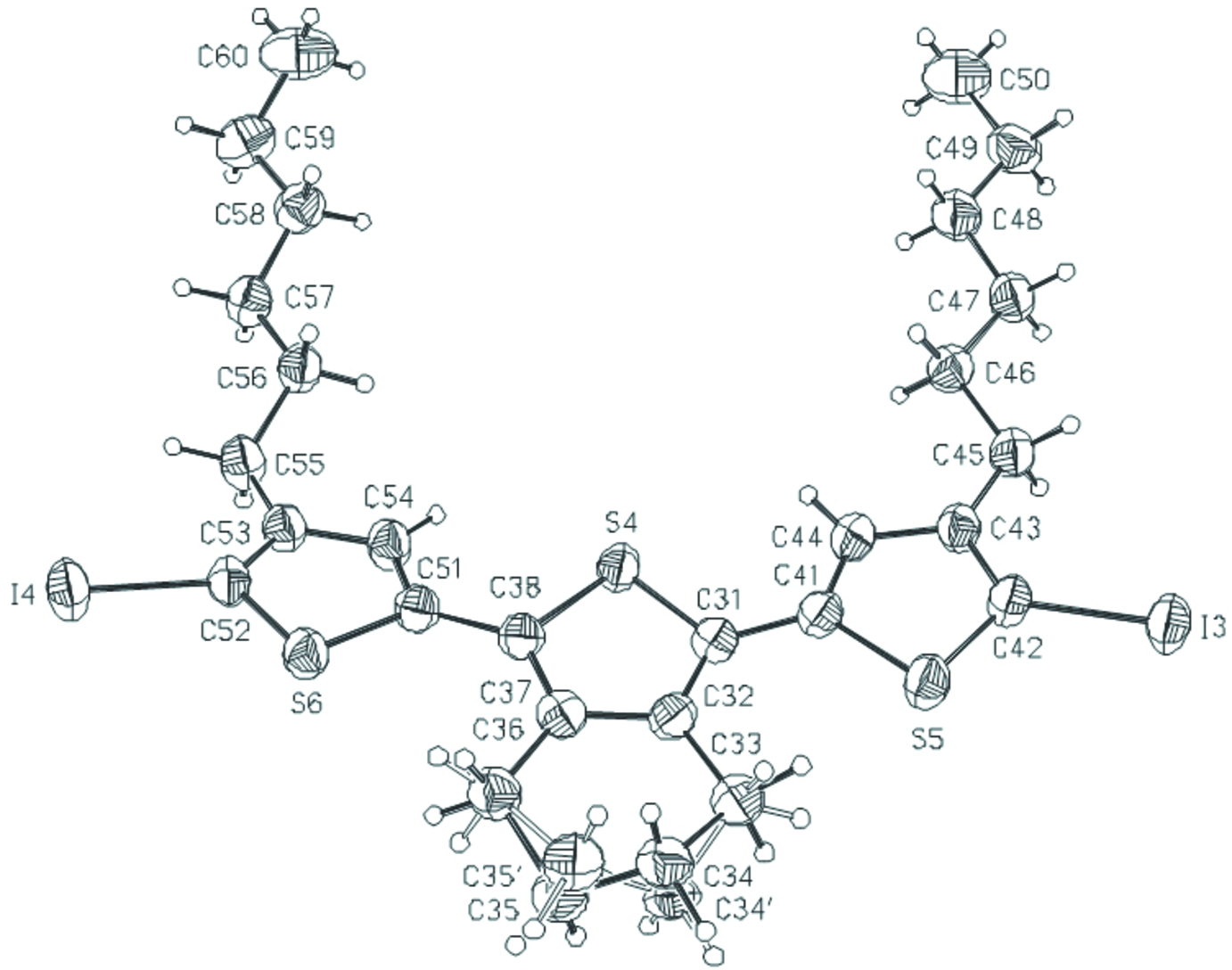

Supplement: Supplementary file 5 [file e-70-o1133-fig2.tif]
